# Are societies becoming more self-centric? Evidence from five decades of popular music spanning three continents

**DOI:** 10.1371/journal.pone.0349765

**Published:** 2026-06-24

**Authors:** Marius Golubickis, Parnian Jalalian, Leoni S. Masroujah, Esther S. Selvaraj, Yadvi Sharma, Siew Hwee Seow, Adriana Borrego-Guerrero, Sadie Marr, C. Neil Macrae

**Affiliations:** 1 Department of Cognitive Sciences, United Arab Emirates University, Al Ain‌‌, United Arab Emirates; 2 School of Psychology, University of Aberdeen, Aberdeen, Scotland‌‌, United Kingdom; 3 School of Social and Health Sciences, James Cook University, Singapore‌‌, Singapore; 4 Institute of Experimental Psychology, University of Regensburg, Regensburg, Germany; Kiel University: Christian-Albrechts-Universitat zu Kiel, GERMANY

## Abstract

A commonly expressed claim is that societal self-centrism has increased over recent decades. To examine this assertion, we analyzed an influential cultural product — popular music — to track changes in self-focus across five decades and three continents. Using an established linguistic analysis technique, personal pronoun usage (i.e., a marker of self-focus) was quantified in the ten most popular songs each year from 1970 to 2019 in the United States, Germany, Japan, and Hong Kong. Results indicated a significant increase in self-focused language over time in individualistic societies (i.e., United States & Germany), whereas no comparable trend was observed in more collectivistic contexts (i.e., Japan & Hong Kong). These findings demonstrate how public artifacts can be leveraged to investigate cultural variation in the expression of self-centrism.

All through the day,

I me mine, I me mine, I me mine

All through the night,

I me mine, I me mine, I me mine.

I me mine – Beatles (1970)

Debates about societal change are a persistent feature of public discourse. Over the past half-century, rapid technological innovation, economic restructuring, and shifting norms have profoundly reshaped social organization and everyday life [1,2]. In turn, these transformations have reconfigured patterns of communication, social interaction, and self-expression in often beneficial ways [3,4]. Yet alongside these developments, concerns have emerged that contemporary societies are increasingly self-focused, as reflected in levels of greed, narcissism, and social disconnection [5–10]. But is this actually the case? Have societies become more self-oriented over the decades — with corresponding intra- and interpersonal consequences — or is this perception overstated? To address this question, we examined data spanning five decades and three continents.

## Cultural products and self-centrism‌‌

Cultural products (e.g., books, TV shows, political speeches) serve as a convenient resource through which putative changes in levels of societal self-centrism can be assessed [11–14]. Broadly speaking, public artifacts signal the characteristics of societies at particular points in time, yielding valuable insights into prevailing cultural norms and the psychological attributes of the population at large (i.e., people are shaped by, and in turn reflect, the cultural context in which they are embedded; [15–18]). At least in Western societies, with emphasis placed on individual rather than collective attainment [19,20], cultural forces are believed to have precipitated increasing levels of self-centrism. As overlapping concepts, individualism and self-centrism both emphasize self over the group. While individualism represents a broader cultural orientation, self-centrism reflects a cognitive dimension of this perspective. An ingenious way in which self-centrism has been examined is by tallying the incidence of first-person singular (e.g., I, mine) compared to first-person plural (e.g., we, us) pronouns — a reliable marker of self-focus [21,22] — in popular cultural products. For example, in American books published between 1960 and 2008, whereas first-person singular pronouns increased in occurrence by 42%, first-person plural pronouns declined by 10% [14]. Over the same period, individualistic words (e.g., “unique”) and phrases (e.g., “all about me”) similarly increased in usage in these tomes [23]

Reflecting its reach, appeal, and cultural significance [24], popular music has proved a valuable repository of information for tracking societal changes in self-centrism [11,13,25,26]. In a seminal article, DeWall and colleagues [13] examined the ten most popular songs each year (based on the Billboard Hot 100 year-end chart) in the United States (US) from 1980 to 2007. Also using pronoun usage to index self-centeredness [14], the results were informative. Across the timeframe of interest (i.e., 28 years), year was positively associated with the use of first-person singular pronouns and negatively associated with the use of first-person plural pronouns, a pattern indicative of rising levels of societal self-centrism [27]. Interestingly, this change aligned with reported increases in narcissistic personality traits in the US [28,29]. As DeWall et al., (2011, p. 206) [13] remarked, “…simply tuning into the most popular songs on the radio may provide people with increased understanding of their generation’s current psychological characteristics.”

Developing this work, Bianchi (2016) [11] explored the relationship between self-centrism and economic prosperity. Specifically, from 1980 to 2014, pronoun usage in popular music in the US was considered alongside annual unemployment rates. The results demonstrated an association between self-focus and the labor market. Whereas popular music was more likely to contain first-person singular pronouns when unemployment rates were low, plural pronouns increased in frequency when unemployment rates were high. In other words, when economic times were good (vs. bad), American pop songs were more focused on self and less centered on others. Confirming the utility of popular music as a marker of societal change ([13,25,26], these findings indicate that, in an individualistic cultural milieu, self-centrism swells during times of prosperity. Importantly, this further highlights that popular music can function as a psychological barometer, subtly reflecting levels of societal self-centeredness at different points in time [15–18].

Notwithstanding the significant insights that have been gleaned from the linguistic analysis of popular music, important questions remain. Prominent among these is the issue of whether increases in self-centeredness represent a worldwide phenomenon or an outcome restricted to Western (i.e., individualistic) societies [30,31]? That is, it is unclear what role culture may play in shaping the lyrics of songs that achieve widespread appeal [16,17]. A distinguishing feature of research to date is that it has focused almost exclusively on music sampled from countries in the West; specifically, the US, United Kingdom (UK), and Canada [11,13,25,26,32]. In each of these nations, subtle differences aside, self-centrism has been shown to be increasing over the decades (i.e., from the late 20^th^ to early 21^st^ centuries), prompting consternation and debate. What has not yet been considered, however, is whether a comparable trend has emerged elsewhere in the world, particularly in non-Western (i.e., collectivistic) societies.

Recent cross-cultural research suggests a measurable rise in individualistic values within historically collectivistic societies, driven by urbanization, economic development, and increased exposure to global media [7–10]. It is possible therefore that, paralleling the pattern observed in the West [11,13,25,26,32], self-centrism (indexed via popular music) has been on the rise everywhere. Alternatively, it is also conceivable that deeply rooted cultural differences (e.g., socialization practices) have exerted a modulatory influence on expressions of self-focus, yielding different temporal profiles in collectivistic and individualistic societies [19,20]. Evidence supporting the latter viewpoint can be garnered from meta-analytic work indicating that socialization practices strongly impact cultural products, including books, press coverage, and magazine/television adverts [16,17]. Morling and Lamoreaux [16], for example, reported that public artifacts from the West (e.g., US, Germany, UK) are both more individualistic and less collectivistic than comparable cultural products from regions in Asia (e.g., India, Hong Kong, Japan). Analogous differences may similarly emerge when examining levels of self-centrism in popular music.

## The Current Research

To provide a comprehensive analysis of potential societal differences in self-centrism, here we examined fifty years of popular music crossing three continents. Covering the period 1970–2019, lyrics were obtained for the ten most popular songs each year from three countries (i.e., US, Germany, Japan) and one Special Administrative Region (i.e., Hong Kong). In so doing, pop songs were gathered from relatively individualistic (i.e., US, Germany) and collectivistic (i.e., Japan, Hong Kong) societies [16,17]. Following previous research, self-centrism was established by probing the linguistic contents of the songs; specifically, the incidence of first-person singular (e.g., I, me, mine) pronouns [11,13]. The relevant data were extracted from the songs using the Linguistic Inquiry Word Count (LIWC) program [33]. Adoption of this approach enabled us to investigate shifts in societal self-centrism as a function of the cultural milieu in which songs achieved prominence [16,17].

## Method

### Pop charts

The pop chart information was curated using both coded web-scraping algorithms and manual extraction from various online and archival databases. Where possible, the information was abstracted for all years from one chart in each country/region. For each country/region, charts were selected based on their representativeness and consistency across time. In this way, the Billboard Hot 100 single end-of-year charts were employed for the US, the Official German Charts for Germany, and the Oricon Single Chart for Japan. For Hong Kong, no single consecutive chart was available for the period of interest (i.e., 1970–2019). As such, from 1970 to 2009 several charts were used (e.g., RTHK Top Ten Chinese Gold Songs Award, JSG Billboard). From 2010 to 2019, the Ultimate Song Chart Award Presentations were employed.

### Language dictionaries

To extract the linguistic contents of the song lyrics, the LIWC program [33] was used, an approach that has been adopted in previous research of this kind. Usefully, the program provides a coding system through which various topics/themes can be abstracted from songs based on the occurrence of particular words. Of relevance to the current inquiry is the frequency of pronoun use (i.e., proportion of pronouns present in a song relative to the overall word count). Based on prior work [11,13], emphasis was placed on first-person singular pronouns (e.g., I, me, myself), but consideration was also given to first-person plural (e.g., we, us, our) and third-person singular/plural pronouns (e.g., he, she, they).

Given the availability of dictionaries in various languages [34–39], songs were analyzed in their original tongue. For each chart, songs were separated based on the language used. Pronouns were extracted using each of the contributory languages (e.g., English & Spanish) and combined for subsequent analysis. The diversity of languages within each chart varied considerably. Whereas the US charts comprised only songs in English, the German charts included songs in German, English, French, Italian, and Spanish. The Japanese charts included songs in Japanese, English, and Chinese, while the Hong Kong charts had songs in Chinese, English, and Japanese.

## Results

### Mixed linear modeling

Mixed linear modeling was adopted to analyze pronoun use across the 50-year time span (2000 songs, 391,078-word corpus). This analytic technique is advantageous for tracking linear changes over time while accounting for other potentially random factors which could influence the contents of popular music [40]. The most comprehensive models of convergence are presented here, incorporating fixed effects of Time, Culture, and Country/Region and random effects of Artist/Song Information, Language Used, and Chart Position (i.e., position in the top 10). Attempts were made to account for differences in chart origin and counting approaches by incorporating when digital media was introduced [13], but these models failed to converge.

#### First-person singular pronouns.

**Fig 1:**
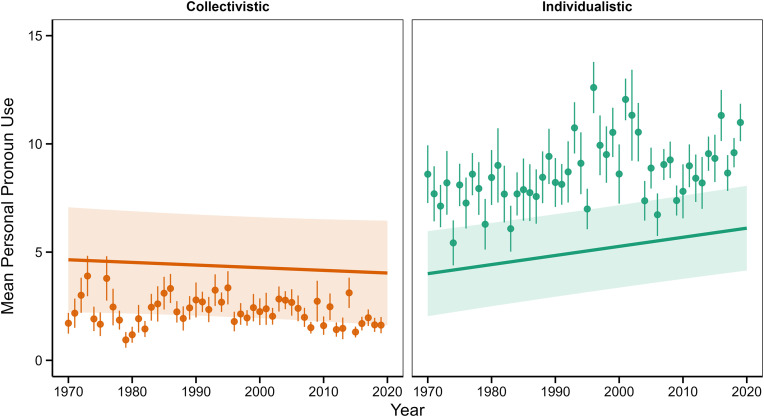
Mean proportion of the first-person singular pronouns used in songs as a function of Year and Culture. Note. Lines represent model predictions (incl. random effects) with 95% Confidence intervals; points represent yearly means ± SE.

The current data yielded evidence that self-centrism has increased over the period under investigation (i.e., 1970−2019), but only in individualistic societies (see Fig 1). Mixed linear modeling confirmed this observation. Our first model tracked the difference between individualist and collectivist cultures in the use of first-person singular pronouns across the temporal window of interest, while controlling for Chart Origin, Language, Song, Artist, and Chart Position. The model revealed a main effect of Culture (*F* = 10.78, *p* < .001) and a significant Year X Culture interaction (*F* = 10.95, *p* < .001). Simple effects showed that the use of personal singular pronouns increased across the 50 years in individualistic (*β* = .042, 95% CI [.023,.061], *t* = 4.37, *p* < .001), but no*t* in collectivistic (*β* = −.012, 95% CI [−.039,.014], *t* = -0.92, *p* = .360) cultures. Additionally, these pronouns occurred more frequently in individualistic compared to collectivistic contexts (*β* = −.054, 95% CI [.087,.022], *t* = 3.30, *p* < .001).

**Fig 2:**
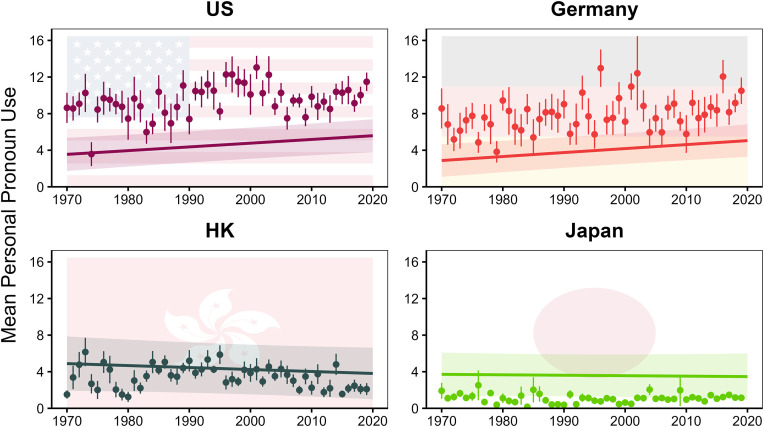
Mean use of first-person singular pronouns as a function of Year and Country/Region. Note. Hong Kong is abbreviated to HK. Lines represent model predictions (incl. random effects) with 95% Confidence intervals; points represent yearly means ± SE.

To explore differences in self-centrism as a function of Chart Origin, our next model incorporated differences in the use of first-person singular pronouns across the four different Countries/Region and Year, while again controlling for Language, Song, Artist, and Chart Position. The model confirmed the previous findings, yielding a significant main effect of Country/Region (*F* = 3.76, *p* = .011) and a Country/Region X Year interaction (*F* = 3.78, *p* = .010, see Fig 2). This model had the best fit (AIC = 10619), indicating the importance of modeling country/region differences (see S1 File (SI): Model reduction). Comparisons (FDR corrected) between the charts showed a significant difference between Germany and Hong Kong (*β* = .065, 95% CI [.002,.128]), *t* = 2.73, *p* = .027) and the US and Hong Kong (*β* = −.062, 95% CI [−.125,.000], *t* = −2.61, *p* = .027), but no reliable difference between Germany and the US (*β* = .003, 95% CI [−.040,.046], *t =* 0.17*, p* = .866) or Japan and Hong Kong (*β* = −.017, 95% CI [−.088,.055], *t* = −0.62, *p* = .645). Japan displayed a smaller reduction in the use of first-person singular pronouns compared to Hong Kong, with the comparison between Japan and Germany (*β* = .048, 95% CI [−.009,.106], *t* = 2.22, *p* = .053), as well as Japan and the US (*β* = −.046, 95% CI [−.103,.012], *t* = −2.09, *p* = .055), failing to reach significance. Examining the trends in personal singular pronoun use across the period of interest for each country revealed a significant increase in the US (*β* = .041, 95% CI [.009,.072], *t* = 3.24, *p* = .003) and Germany (*β* = .043, 95% CI [.012,.075], *t* = 3.46, *p* = .002), but not in Japan (*β* = −.005, 95% CI [−.050,.040], *t* = −0.28, *p* = .783) or Hong Kong (*β* = −.022, 95% CI [−.072,.029], *t* = −1.06, *p* = .383).

#### Other pronouns.

No significant Year X Culture interactions were observed for the use of first-person plural, third-person singular, or third-person plural pronouns (see SI Comparisons with other pronouns, Table SI-1 and Figure SI-1 in S1 File).

## Discussion

Examining word use in popular song lyrics across five decades and three continents, we explored whether societal self-centrism has changed over time. The findings both replicated and extended prior work on the topic [11,13,25,26]. Corroborating previous research, first-person singular pronoun usage increased in the US between 1970 and 2019 [11,13], consistent with rising self-centrism. A comparable pattern was observed in Germany, suggesting increases in self-referential language may characterize Western, individualistic societies more broadly [9]. Increasing self-centrism did not generalize across cultures, however. In more collectivistic settings — specifically Hong Kong and Japan — first-person singular pronouns appeared less frequently in song lyrics than in the US and Germany and displayed no increase over the decades. These findings indicate that temporal changes in self-centrism are culturally contingent rather than universal, aligning with evidence that public artifacts (e.g., advertisements, books) from East Asian countries tend to exhibit lower levels of self-focus than those from the West [16,17].

Contrasting DeWall et al. [13] who reported a decline in the use of first-person plural pronouns (e.g., we, us) in popular music, no comparable trend was observed in the current investigation. That is, in the US and Germany, increased self-centrism over the decades was not accompanied by a concomitant reduction in focus on others. Two factors may account for this divergence. First, compared to DeWall et al. [13], the current research covered a larger temporal window (i.e., 1980–2007 vs. 1970–2019), potentially capturing longer-term shifts in pronoun use. Second, conceptualized as orthogonal dimensions, changes on one dimension do not necessarily entail inverse changes in the other, particularly given their sensitivity to operationalization and measurement [17]. Also, Bianchi (2016) [11] reported that changes in pronoun use were not linear over time, but instead fluctuated in response to broader socio-economic conditions. This non-linearity is relevant to the current work, as it suggests that observed increases (or stability) in self-centrism across cultures may mask shorter-term variability tied to contextual factors (e.g., economic cycles). As such, the present findings may reflect not only enduring cultural differences but also the aggregation of dynamic, context-sensitive shifts in pronoun use across the 50-year period under investigation.

Although the current findings were derived from the analysis of a single product —popular songs —the psychological and cultural significance of music is incontestable [24,41], particularly (though not exclusively) among adolescents and young adults [42,43]. Estimates suggest that young adults listen to an average of 20.7 hours of music per week, with many reporting benefits for wellbeing, coping, and stress management [44–46]. Additionally, engagement with popular music contributes to identity development and self-definition, as music serves as a conduit through which people can signal their values, group memberships, and ideologies [41,47]. In a wider context, popular music also serves as a socializing agent, normalizing attitudes to gender roles, interpersonal relationships, and mental health by exposing listeners to shared themes and dominant societal representations [48]. These psychological influences are amplified through mass media and digital platforms, enabling popular music to shape collective experiences and contribute to broader cultural norms and belief systems.

Given the well-established influence of culture on both individual and collective behavior, the divergent trajectories of self-centrism observed between Western and East Asian societies warrants closer examination. Although some research has suggested that parts of Asia have undergone shifts toward greater individualism [7–10,49–51], the overall pattern remains mixed and far from conclusive. For instance, work in China has provided converging evidence for rising individualism across several behavioral and linguistic domains. Indicators include an increased preference for uniqueness, reflected in more distinctive baby names and shifts in language use — most notably, a greater prevalence of first-person singular pronouns in written texts [52,53]. At the same time, however, other linguistic analyses challenge this narrative. Using alternative corpora and analytic approaches, Hamamura et al. [54] reported little consistent evidence for increasing individualism in China over a fifty-year period (i.e., 1950–1999). Taken together, these findings suggest that claims of rising individualism in East Asian societies may be overstated. Rather than a uniform cultural shift, the available evidence points to a more nuanced process characterized by domain-specific changes and the potential coexistence of individualistic and collectivistic tendencies [16,17].

The current results side with this interpretation. Whereas self-focused language increased markedly in the US and Germany between 1970 and 2019, no comparable rise was observed in Japan and Hong Kong. Instead, starting from a lower baseline, self-centrism in these East Asian contexts remained relatively stable across the decades. This pattern is consistent with previous meta-analytic evidence indicating that cultural products systematically differ as a function of individualism-collectivism [16,17]. Across domains such as internet contents, books, and press coverage, cultural artifacts originating in East Asia tend to express lower levels of individualism than those produced in Western societies. As indicated by the current results, popular music appears to follow a similar pattern. These findings imply that although societal self-centrism may be increasing in some regions and on certain indices [9], such shifts are neither uniform nor consistently reflected in expressive cultural artifacts.

The observed societal differences in self-centrism are noteworthy in no small part because heightened self-focus has been linked to a range of maladaptive traits and behaviors. One such trait is narcissism, which is characterized by antagonism, grandiosity, and elevated self-centrism [55]. Consistent with this characterization, individuals scoring higher on narcissistic traits use first-person singular pronouns more frequently than others, reflecting increased levels of self-focus [28]. Narcissistic tendencies are also associated with the endorsement and expression of individualistic values [56]. Accordingly, increases in individualism observed in countries such as the US and Germany over recent decades [57] have been accompanied by corresponding increases in self-centrism (indexed via personal pronoun use), as confirmed by the current findings. Beyond narcissism, greed represents another construct linked to heightened egocentrism and individualism. Defined as a strong desire for excessive accumulation, greed is associated with outcomes that can be detrimental to others. For example, higher levels of greed predict selfish economic decisions that prioritize personal gain over collective welfare [58], a pattern that allies with the broader association between individualism and self-oriented behavior [59].

The current investigation has several limitations that merit attention. First, although popular songs provide a valuable and accessible source for examining psychological constructs across time, they represent only one type of cultural product with this potential. Digitized cultural artefacts — most notably books — would unquestionably offer complimentary insights into psychological qualities across societies and historical periods [60,61]. As books extend much further back in history than many other products, they may be particularly well suited for investigating long-term changes in self-centrism. Second, while analyzing song lyrics in their original languages afforded several methodological advantages, an alternative approach would have been to translate all lyrics into a single language (e.g., English) prior to text analysis. Each strategy involves distinct trade-offs. Native-language analysis preserves language-specific semantic subtleties but may be less sensitive to cross-linguistic grammatical differences. In contrast, translation-based approaches may reduce issues of structural heterogeneity across languages, but at the cost of diminished linguistic nuance and semantic fidelity [62]. Future research should therefore explicitly consider these trade-offs when selecting analytic strategies for cross-cultural text analysis. Third, given the historical and cross-cultural scope of the dataset, consistent and reliable classification of musical genre across all regions and decades was not feasible. As such, future research may benefit from incorporating genre-sensitive analyses where relevant data are available.

## Conclusion

The present findings revealed that societal increases in self-centrism are neither inevitable nor universal [11,13,25,26] but instead depend on broader cultural value systems [16,17]. By tracking first-person singular pronoun use in popular song lyrics across five decades and multiple regions, our research highlights how expressive cultural products can reflect — and potentially reinforce — dominant forms of self-construal. Whereas Western societies exhibited a clear increase in self-focused language over time, East Asian societies showed relative stability, underscoring the enduring influence of collectivistic norms on the public expression of self-centrism [16,17]. These results contribute to a body of evidence suggesting that shifts toward individualism unfold unevenly across societies and are expressed selectively across domains, highlighting the importance of culturally sensitive interpretations of global trends.

## Supporting information

S1 File Supporting information. Additional methodological details, analyses, tables, and figures supporting the findings reported in this manuscript.
(DOCX)
