# Supplementary material for: Are societies becoming more self-centric? Evidence from five decades of popular music spanning three continents
Source: PLoS One. 2026 Jun 24;21(6):e0349765. doi: 10.1371/journal.pone.0349765 (PMC13293456; doi:10.1371/journal.pone.0349765)

**SUPPORTING INFORMATION**

Are societies becoming more self-centric?

Evidence from five decades of popular music spanning three continents

Marius Golubickis,^1^ Parnian Jalalian,^2^ Leoni S. Masroujah,^2^ Esther S. Selvaraj,^2^ Yadvi Sharma,^2^

Siew Hwee Seow,^3^ Adriana Borrego-Guerrero,^4^ Sadie Marr,^2^ C. Neil Macrae^2^

^1^Department of Cognitive Sciences, United Arab Emirates University, Al Ain, UAE

^2^School of Psychology, University of Aberdeen, Aberdeen, Scotland, UK

^3^School of Social and Health Sciences, James Cook University, Singapore

^4^Institute of Experimental Psychology, University of Regensburg, Regensburg, Germany

This document includes:

- SI text section 1-2
- SI Table 1
- SI Figure 1

**SI text section**

1. **Model-reduction**

Model comparisons revealed the significant amount of variance the fixed effects accounted for (see Table SI-1). The best-fitting model with the lowest AIC (AIC = 10619) modeled the interaction between the various countries/regions and year. In comparison, the model which took into account cultural differences more broadly (i.e., individualist vs collectivist) showed a lower model fit (AIC = 10625). Model fit dropped further as the fixed effects were reduced, and while the random effects showed a significant contribution to variance, the model fit remains lower. These comparisons confirm that the fixed effects explain important variance in pronoun use above and beyond the variance that the random factors account for. Hence, suggesting the importance of year and country/region in the personal pronoun use in popular music.

1. **Comparison with other pronouns**

Further comparisons were carried out between first-person plural pronoun use and third-person pronoun use. Modelling first-person plural pronouns (e.g., we, us, our) against country/region and year only showed a main effect of Year, *F* = 4.422, *p* = .035, and no significant effect of Country, *F* = 1.991, *p* = .120, nor interaction between the two factors, *F* = 2.041, *p* = .113, see Figure SI-1a. Note that for convergence reasons, only song and artist information were modeled as random effects. Models for third-person singular and plural pronouns similarly revealed no interaction between country/region and year. The model for third person singular pronouns had no significant effects (Country: *F* = 0.150, *p* = .930, Year: *F* = 3.522, *p* = .061, Year X Country: *F* = 0.135, *p* =.939, see Figure SI-1b) nor did the model for third person plural pronouns (Country: *F* = 0.237, *p* = .870, Year: *F* = 0.823, *p* = .364, Year X Country: *F* = 0.274, *p* =.845, see Figure SI-1c). Note that the third-person plural pronoun model took into account the full random structure, while the third-person singular pronoun model dropped the chart place as a random factor due to convergence. These results confirm the unique and important development of self-centric language in pop songs.

**SI Table 1:** The table details the two main MLM models, which are discussed above and model culture and country/region separately. The table further details model reductions to determine the best model fit. Each model is compared to the model in the line above. A lower AIC value indicates a better model fit. Chi-square significance comparison shows which of the two models explains more variance, with the complex model being better when indicated by the (*).

|  | **Model** | **AIC** | **Significance** |
| --- | --- | --- | --- |
| Culture Model | i ~ year*culture + (1 country:language) + (1 \| song ) + (1 \| artist ) + (1 \| chart place ) | 10625 |  |
| Country Model | i ~ year*country + (1 \|language) + (1 \| song ) + (1 \| artist ) + (1 \| chart place ) | 10619 | Chisq = 13.433, p = .009 |
| Additive Model | i ~ year*county + (1 \|language) + (1 \| song ) + (1 \| artist ) + (1 \| chart place ) | 10625 | Chisq = 11.282, p = .01 – country model* |
| Country only | i ~ country + (1 \|language) + (1 \| song ) + (1 \| artist ) + (1 \| chart place ) | 10632 | Chisq = 8.959, p = .003 – additive model* |
| Random only | i ~ 1 + (1 \|language) + (1 \| song ) + (1 \| artist ) + (1 \| chart place ) | 10631 | Chisq = 5.770, p = .123 |
| Drop langauge | i ~ 1 + (1 \| song ) + (1 \| artist ) + (1 \| chart place ) | 11299 | Chisq = 669.48, p < .001 – random only* |
| Drop Song | i ~ 1 + (1 \| artist ) + (1 \| chart place ) | 11329 | Chisq = 32.602, p < .001 – language model* |
| Drop artist | i ~ 1 + (1 \| chart place ) | 11836 | Chisq = 509.11, p < .001 – artist model* |

**SI Figure 1:** The figure combines the model graphs for the models for first-person plural pronouns (part a), third-person singular pronouns (part b), and third-person plural pronouns (part c).


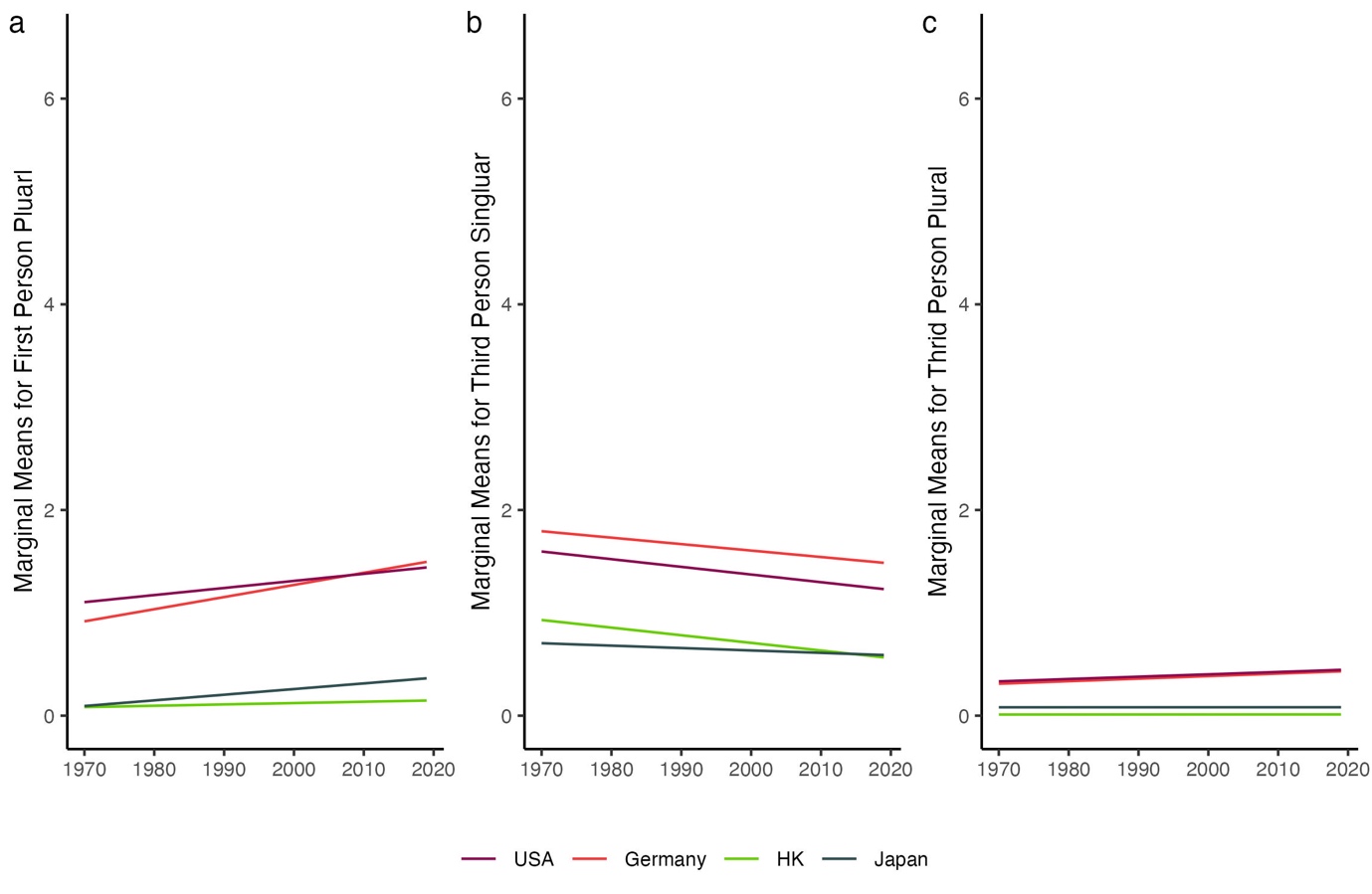

Supplement: S1 File — Additional methodological details, analyses, tables, and figures supporting the findings reported in this manuscript. (DOCX) [file pone.0349765.s001.docx]
